# Hemispheric Differences in Functional Interactions Between the Dorsal Lateral Prefrontal Cortex and Ipsilateral Motor Cortex

**DOI:** 10.3389/fnhum.2020.00202

**Published:** 2020-06-03

**Authors:** Yanqiu Wang, Na Cao, Yitong Lin, Robert Chen, Jian Zhang

**Affiliations:** ^1^School of Psychology, Shanghai University of Sport, Shanghai, China; ^2^Krembil Research Institute, University Health Network, Toronto, ON, Canada; ^3^Division of Neurology, Department of Medicine, University of Toronto, Toronto, ON, Canada; ^4^Department of Life Sciences, Graduate School of Arts and Sciences, University of Tokyo, Tokyo, Japan

**Keywords:** dorsolateral prefrontal cortex, primary motor cortex, functional connectivity, hemispheric differences, transcranial magnetic stimulation

## Abstract

**Background:** The dorsolateral prefrontal cortex (DLPFC) in both hemispheres have a central integrative function for motor control and behavior. Understanding the hemispheric difference between DLPFC and ipsilateral motor cortex connection in the resting-state will provide fundamental knowledge to explain the different roles DLPFC plays in motor behavior.

**Purpose:** The current study tested the interactions between the ipsilateral DLPFC and the primary motor cortex (M1) in each hemisphere at rest. We hypothesized that left DLPFC has a greater inhibitory effect on the ipsilateral M1 compared to the right DLPFC.

**Methods:** Fourteen right-handed subjects were tested in a dual-coil paired-pulse paradigm using transcranial magnetic stimulation. The conditioning stimulus (CS) was applied to the DLPFC and the test stimulus (TS) was applied to M1. Interstimulus intervals (ISIs) between CS and TS were 2, 4, 6, 8, 10, 15, 20, 25, and 30 ms. The result was expressed as a percentage of the mean peak-to-peak amplitude of the unconditioned test pulse.

**Results:** There was stronger inhibitory effect for the left compared to the right hemisphere at ISIs of 2 (*p* = 0.045), 10 (*p* = 0.006), 15 (*p* = 0.029) and 20 (*p* = 0.024) ms. There was no significant inhibition or facilitation at any ISI in the right hemisphere.

**Conclusions:** The two hemispheres have distinct DLPFC and M1 cortico-cortical connectivity at rest. Left hemisphere DLPFC is dominant in inhibiting ipsilateral M1.

## Introduction

The dorsolateral prefrontal cortex (DLPFC) has a central integrative function for motor control and behavior (Miller and Cohen, 2001; Cieslik et al., 2013). In particular, DLPFC has diverse connections to several different motor regions such as the premotor cortices, supplementary motor area, cerebellum, and basal ganglia (Alexander et al., 1986; Bates and Goldman-Rakic, 1993; Lu et al., 1994; Miller and Cohen, 2001). Previous imaging studies have found differences in cortical connections between the left and right hemispheres, including the connection between PFC and motor area (Tian et al., 2011; Daianu et al., 2012). Although there are no direct anatomical connections between DLPFC and primary motor cortex (M1; Miller and Cohen, 2001), research using transcranial magnetic stimulation has indicated coupling between DLPFC and M1 in the millisecond timescale (Duque et al., 2012).

Previous studies have assigned different roles in motor control to the left and right DLPFC (Rubia et al., 2001; Fierro et al., 2010; Kantak et al., 2010; Jin et al., 2019). The left DLPFC was associated with force control (Jin et al., 2019), motor memory maintenance (Kantak et al., 2010), and motor inhibition (Rubia et al., 2001). The right DLPFC was found to influence ocular motor behavior (Pierrot-Deseilligny et al., 2005) and showed decreased regional cerebral blood in Parkinson’s disease (Kikuchi et al., 2001). Moreover, the interaction between DLPFC in the two hemispheres may contribute to motor behavior since increasing the excitability of the right DLPFC and inhibition of the left DLPFC enhanced behavioral planning (Heinze et al., 2014). However, tasks that required brain activation confound the fundamental cortico-cortical connectivity between DLPFC and ipsilateral M1. Therefore, testing the resting-state functional connectivity between DLPFC and ipsilateral M1 can provide basic knowledge of their connections.

With a conditioning coil placed over a non-M1 area and a test coil over the hand area of the M1 (M1HAND), dual-site transcranial magnetic stimulation (dsTMS) has been established as a valuable tool to probe the excitability of the cortico-cortical inputs from ipsilateral and contralateral frontal areas to the M1HAND at rest (Civardi et al., 2001; Mochizuki et al., 2004; Koch et al., 2007; Bäumer et al., 2009; Ni et al., 2009). These studies found inhibitory or facilitatory interactions depending on the dsTMS protocol (Koch et al., 2007; Davare et al., 2008). The interactions critically depend on the timing of the conditioning stimulus (CS) relative to the test stimulus (TS).

Previous studies reported hemispheric asymmetry motor cortex excitability at rest and during unimanual motor tasks (Netz et al., 1995; Ziemann and Hallett, 2001). Greater inhibitory effects from the left to the right motor cortex than vice versa were reported (Netz et al., 1995). Moreover, the left motor cortex was more active during ipsilateral hand movements, suggesting that there was less inhibition from the active right compared to the active left motor cortex (Ziemann and Hallett, 2001). Although both studies concluded that the left hemisphere is the dominant motor cortex, most studies examined interhemispheric connections (Mochizuki et al., 2004; Ni et al., 2009), and few studies tested connections between different cortical areas in the same hemisphere (Koch et al., 2007; Brown et al., 2019). The study of hemispheric differences in the ipsilateral DLPFC-M1 connection will enhance our understanding of hemispheric differences in connections with the motor cortex.

On such grounds, the current study used dsTMS to test the interactions between the ipsilateral DLPFC and M1 at rest in both hemispheres. We hypothesized that left DLPFC will have greater inhibitory effects on the ipsilateral M1 compared to the right DLPFC.

## Materials and Methods

### Subjects

Fourteen right-handed subjects participated in the experiment. Handedness was assessed using the Oldfield Handedness Inventory (Oldfield, 1971). All subjects provided written informed consent following the Declaration of Helsinki. The protocol was approved by the Shanghai University of Sports Research Ethics Board.

### Electromyogram (EMG) Recording

EMG was recorded from the first dorsal interosseous (FDI) muscles in both hands using 9 mm diameter Ag-AgCl surface-cup electrodes. The active electrode was placed over the muscle belly and the reference electrode over the metacarpophalangeal joint of the index finger. The signal was amplified (1,000×), band-pass filtered (2 Hz–2.5 kHz, Intronix Technologies Corporation Model 2024F, Bolton, ON, Canada), digitized at 5 kHz by an analog-to-digital interface (Micro1401, Cambridge Electronics Design, Cambridge, UK) and stored in a computer for off-line analysis using SIGNAL software (Cambridge Electronic Devices, Cambridge, UK).

### Transcranial Magnetic Stimulation

**Figure 1:**
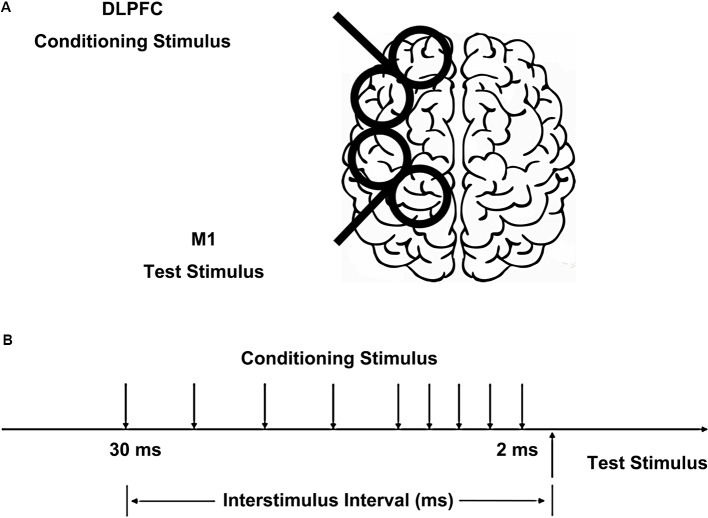
Placement of transcranial magnetic stimulation (TMS) coils and stimulus configurations. **(A)** The conditioning coil was placed on the dorsolateral prefrontal cortex (DLPFC) and the test coil was placed on the primary motor cortex (M1). **(B)** The interstimulus intervals (ISIs) between conditioning stimulus (CS) and test stimulus (TS) were 2, 4, 6, 8, 10, 15, 20, 25, and 30 ms.

We used a paired-pulse stimulation technique with two high-power Magstim200 machines (Magstim, Whitland, UK). The hotspot of M1 was defined as the scalp location that induced the largest peak to peak motor-evoked potential (MEP) amplitude in the contralateral FDI muscle. The intensity of the test stimulus (TS) was adjusted to evoke MEP of 1 mV peak-to-peak in the relaxed FDI muscle using a figure-of-eight shaped coil (40 mm Alpha Branding Iron, Magstim). The handle of the coil pointed backward at 45° from the mid-sagittal line to induce a posterior-anterior directed current in the brain. We defined the resting motor threshold (RMT) as the lowest intensity that evoked at least five small responses (>50 μV) in the contralateral FDI muscle in a series of 10 stimuli when the subject kept the FDI muscles relaxed in both hands according to international standards (Rossini et al., 2015). A previous study has shown CS intensities of 80% and 120% RMT on DLPFC had similar effects on M1 in both the right and left hemispheres (Brown et al., 2019). Therefore, the CS on DLPFC was set at 110% of RMT using the same type of coil as the TS, placed at 5 cm anterior to the FDI hotspot (Ni et al., 2009), inducing a current in the anterior to posterior direction (Figure 1). Based on the study of Ni et al. (2009), different CS current directions on DLPFC had similar effects on contralateral M1. The TS and CS intensities were determined separately for the left and right hemispheres. The interstimulus intervals (ISIs) between CS and TS were 2, 4, 6, 8, 10, 15, 20, 25, and 30 ms. The 10 conditions were randomly intermingled: TS alone (MEP) and CS plus TS at nine different ISIs. Twenty trials were tested for the test stimulus alone and 10 trials for each ISI. The trials were delivered 5 s apart. In half of the participants, the left hemisphere was tested first and in the other half, the right side was tested first.

### Data Analysis

The peak-to-peak MEP amplitude of each conditioned trial was expressed as a percentage of the mean peak-to-peak MEP amplitude from the unconditioned test pulse. First, to determine whether there was an inhibitory or facilitatory effect at each ISI, the MEP ratios of each ISIs were compared to TS alone in each hemisphere using a paired *t*-test. Second, the effect of DLPFC on ipsilateral M1 (ratio) were analyzed with two-way repeated-measures analysis of variance (ANOVA) using hemisphere (left and right) and ISIs (TS alone and nine ISIs) as within-subject variables. If the interaction effect was significant, it was further explored by comparing the two hemispheres at each ISIs using a paired *t*-test. Mauchly’s test was used to examine for sphericity and the Greenhouse-Geisser correction was used for nonspherical data.

## Results

In the left hemisphere, there was significant inhibition at ISIs of 2 (*p* = 0.012), 10 (*p* = 0.001), 15 (*p* = 0.05) and 20 (*p* = 0.005) ms compared to the TS alone. In right hemisphere there was no significant inhibition or facilitation at any ISI.

**Figure 2:**
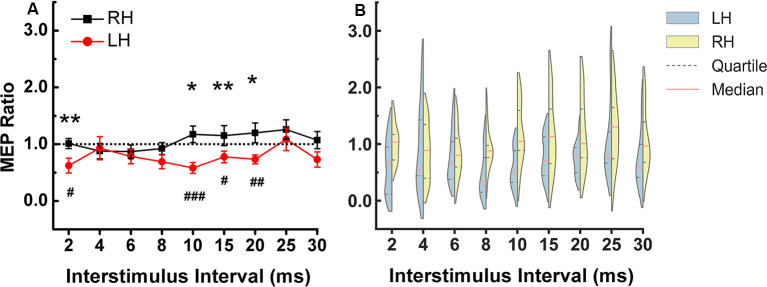
DLPFC to ipsilateral M1 connection at rest. **(A)** The stronger inhibitory effect was observed in the left compared to the right hemisphere at ISIs of 2, 10, 15, and 20 ms. The asterisks indicate the comparisons between two hemispheres at each ISIs (*p < 0.05, **p < 0.01). The pound symbol indicates the ISI in each hemisphere that showed significant inhibition compared to TS alone (^#^*p* < 0.05, ^##^*p* < 0.01, ^###^*p* < 0.001). Errors bars indicate one standard error of the mean. **(B)** Each violin spans the 98% quantile of the motor-evoked potential (MEP) ratio distribution with width proportional to kernel density distributions at each CS-TS interstimulus interval. The red dense dashed lines represent the median and the black sparse dashed lines represent the 25th and 75th percentile. Note: In Figure 2, the means were plotted in **(A)** while the medians and quartiles were plotted in **(B)**. Panel **(B)** shows that for left hemisphere at 4 ms, some data points have values higher than 2 (two subjects have relatively higher MEP ratios), there are some data points in the 1.5–2 range and the rest of the data points are in the range of 0–1.5. Due to the skewed distribution of data points, the mean (0.93) is higher than the median (0.54). In the right hemisphere at 4 ms, the data is close to a normal distribution so the mean (0.88) is similar to the median (0.89). X-axis: interstimulus interval between CS and TS (ms). Y-axis: conditioned MEP amplitude expressed as a ratio to the mean unconditioned MEP amplitude for test pulse alone. Ratios above one indicate facilitation and ratios below one indicate inhibition of the ipsilateral M1. LH, left hemisphere; RH, right hemisphere.

The two-way repeated-measures ANOVA showed no significant main effect for “hemisphere” and “ISIs”, but there was a significant interaction between hemisphere and ISIs (*F* = 2.007, *p* = 0.044). Figure 2 shows that the significant interaction is due to greater DLPFC-M1 inhibition at several ISIs in the left compared to the right hemisphere. Paired *t*-test between the two hemispheres at each ISI showed a stronger inhibitory effect in the left compared to the right hemisphere at ISI of 2 (*p* = 0.45), 10 (*p* = 0.006), 15 (*p* = 0.029) and 20 (*p* = 0.024) ms (Figure 2). All of Mauchly’s tests were not significant.

## Discussion

We examined the hemispheric differences in cortical connectivity between ipsilateral DLPFC and M1 at rest. We found two distinct phases of hemispheric difference in DLPFC-M1 interaction at ISIs of 2 ms and 10, 15, 20 ms. In the left hemisphere, DLPFC stimulation inhibited the M1 excitability at ISIs of 2 ms and 10, 15, 20 ms.

### Dominant Inhibitory Effects of the Left Hemisphere

We found that the left DLPFC has a greater inhibitory effect than the right DLPFC on ipsilateral M1 at a short ISI of 2 ms and longer ISIs from 10 to 20 ms, showing that the DLPFC to M1 connectivity is different in the left compared to the right hemisphere. This is in line with previous studies showing the hemispheric asymmetry of interhemispheric interactions (Ziemann and Hallett, 2001). The left motor cortex exerts greater inhibitory effects on the contralateral motor cortex when the ipsilateral hand performs a movement, compared to the right motor cortex (Ziemann and Hallett, 2001). Interhemispheric inhibition from left hemisphere stimulation was more marked than from the right hemisphere stimulation (Netz et al., 1995). Moreover, inhibitory rTMS modulation of the left DLPFC increased ipsilateral M1 excitability, and facilitation of the left DLPFC by rTMS decreased ipsilateral M1 excitability, which suggested an inhibitory connection from left DLPFC to M1 (Cao et al., 2018). Overall, there is evidence that the left hemisphere showed greater inhibition than the right hemisphere.

Unlike previous studies that focused on prefrontal and motor cortex interactions in the left hemisphere only (Fierro et al., 2010; Jin et al., 2019), we also examined DLPFC-M1 interaction in the right hemisphere and were not able to find that right DLPFC stimulation has an influence on ipsilateral M1 excitability using the 110% RMT CS intensity. Our findings are in line with those of a previous study by Brown et al. (2019), who were also unable to find a causal influence of right-DLPFC on ipsilateral M1 excitability at rest and during sustained isometric using 80% and 120% RMT CS intensity. To more definitively conclude the DLPFC’s influence on ipsilateral M1, future studies should investigate more stimulus intensities over the right and left hemispheres to ensure that CS intensity is optimal for inducing effective influence. Moreover, the findings in the right hemisphere could also be due to the low degree of brain activation in the resting state. Tasks that involve higher-level cognitive processing could also be assessed in future studies to further examine DLPFC-M1 hemispheric differences. Moreover, the DLPFC is a multimodal area involved in several different cognitive functions (Melrose et al., 2007; Cieslik et al., 2013; Kaller et al., 2013; Korgaonkar et al., 2013), such as memory, attention, inhibition, planning, emotional control, and abstract reasoning. The “hotspot” within the DLPFC for these functions may differ among individuals and between hemispheres. This variation could partially account for our findings.

### Inhibitory Pathway in DLPFC to M1 Projection

The peak inhibitory effect from left DLPFC to left M1 was at 10 ms (Figure 2). This is similar to a previous study that reported peak inhibition from left DLPFC to M1 at 12 ms (Hasan et al., 2013). Previous studies showed the posterior parietal cortex potentiates the M1 at an ISI of 4 ms (Koch et al., 2007). CS of 90% RMT intensity on the premotor cortex inhibited M1 and CS of 120% RMT intensity facilitated M1 at ISI of 6 ms (Civardi et al., 2001). Considering the distance and the lack of evidence of direct white matter fibers connection (Guye et al., 2003) between DLPFC-M1, it is likely that the peak inhibitory effect of DLPFC and M1 at about 10 ms is due to indirect connections. A previous study suggested that the frontal cortex indirectly influence the basal ganglia *via* projection to the pre-supplementary motor area (preSMA) and thus influencing M1 through the connectivity between basal ganglia and M1 (Duann et al., 2009).

The pathway mediating the inhibition at the very short 2 ms ISI in the left hemisphere between DLPFC and M1 is not known. This cannot be explained by superior longitudinal fasciculus (SLF) between premotor and M1 which is a large bundle of association fibers in the white matter of each cerebral hemisphere connecting the parietal, occipital and temporal lobes with ipsilateral frontal cortices (Schmahmann et al., 2008), because anatomically closer area such as the premotor area has longer projection time for example 6 ms (Civardi et al., 2001). Future research should examine whether there is a new subcomponent of SLF.

There are several limitations to our study. First, we tested only one CS intensity. A wider range of conditioning stimulation intensities should be tested in future studies and we cannot exclude the possibility that with different CS intensities the right DLPFC-M1 connection will also show significant inhibition. Second, we did not use MRI based neuronavigation system to locate the DLPFC. Previous studies have localized the DLPFC based on distance from the M1 hotspot and achieved reliable results (Civardi et al., 2001; Fierro et al., 2010). However, due to the variation in the gyrification of cortex between individuals, possibly, we stimulated areas that accounted for other cognitive functions using the current coil placement protocol. Moreover, differences in gyral patterns between the left and right hemispheres could lead to stimulation of different parts of the frontal area, although previous studies did not find a systematic hemispheric difference in the gyral pattern in the DLPFC (Toga and Thompson, 2003). Third, we used only one CS current direction. Since the current direction may influence the effects of stimulation, we cannot exclude the possibility that different results may be obtained if different coil orientations are used. Fourth, the contralateral DLPFC-M1 connectivity was not tested. Right DLPFC has been shown to inhibit contralateral M1 at ISIs of 30–60 ms in right-handed subjects (Ni et al., 2009). Further studies are needed to address whether there is a hemispheric asymmetry in the from DLPFC to contralateral M1. Fifth, our sample size is relatively small.

## Conclusion

Our findings suggest that the two hemispheres have distinct DLPFC and M1 cortico-cortical connectivity at rest. Left hemisphere DLPFC is dominant in inhibiting ipsilateral M1.

## Data Availability Statement

The datasets generated for this study are available on request to the corresponding author.

## Ethics Statement

The studies involving human participants were reviewed and approved by Shanghai University of Sports Research Ethics Board. The patients/participants provided their written informed consent to participate in this study.

## Author Contributions

YW, NC, and JZ designed the study. YW, NC, and YL collected the data. YW analyzed the data and wrote the article. RC and JZ reviewed the article.

## Conflict of Interest

The authors declare that the research was conducted in the absence of any commercial or financial relationships that could be construed as a potential conflict of interest.
